# Molecular Characterization of the Effect of Glucagon-Like Peptide-1 Receptor Agonist Semaglutide in the Nephrotoxic Serum Nephritis Mouse Model

**DOI:** 10.34067/KID.0000001067

**Published:** 2025-12-03

**Authors:** Jaime Moreno Martinez, Maria Ougaard, Tanya Grancharova, Regitze Dalsgaard Zdravkovic, Mette Viberg Østergaard, Agnès Bénardeau, Lotte Bjerre Knudsen, Charles Pyke, Peter Helding Kvist, Henning Hvid

**Affiliations:** Research and Early Development, Novo Nordisk A/S, Denmark

**Keywords:** CKD, diabetes, diabetic nephropathy, gene expression, obesity

## Abstract

**Key Points:**

- Treatment with semaglutide improves kidney function and pathology in the mouse nephrotoxic serum nephritis model.
- Transcriptomics data demonstrated that semaglutide had anti-inflammatory and antifibrotic effects and indicated beneficial effects on renal hemodynamics.
- These beneficial effects of semaglutide occur independent of metabolic effects.

**Background:**

CKD is a significant public health issue, affecting approximately half a billion people globally. Key risk factors for CKD include obesity, hypertension, cardiovascular diseases, and diabetes. Glucagon-like peptide-1 receptor agonists are effective treatments for obesity and diabetes. The FLOW trial recently showed that treatment with the glucagon-like peptide-1 receptor agonists semaglutide significantly reduced the incidence of clinically important kidney outcomes in patients with type 2 diabetes and CKD, likely through beneficial effects on kidney blood flow, inflammation, and fibrosis as well as effects mediated by improvement of glycemic control. This study aimed to characterize the effects of semaglutide in the mouse nephrotoxic serum nephritis model, a nonobese and nondiabetic mouse model of CKD.

**Methods:**

Mice were treated with semaglutide or the angiotensin-converting enzyme inhibitor enalapril for 14 days. Various kidney function parameters were measured, and gene expression in key kidney compartments was explored using spatial transcriptomics and single-nucleus RNA sequencing in kidney samples collected at the end of the study.

**Results:**

Semaglutide treatment significantly improved kidney function parameters and changed the expression of multiple genes involved in inflammatory processes and fibrosis, such as *Spp1*, and also affected gene expression in the renin-angiotensin-aldosterone system. These findings from spatial transcriptomics were validated by histology, which also revealed that semaglutide decreased mesangial expansion and had a beneficial effect on filtration slit density in the glomeruli.

**Conclusions:**

These findings demonstrate that the beneficial effects of semaglutide treatment in this rodent model of CKD can occur separately from its antiobesity and antidiabetes effects.

## Visual Abstract

**Figure:**
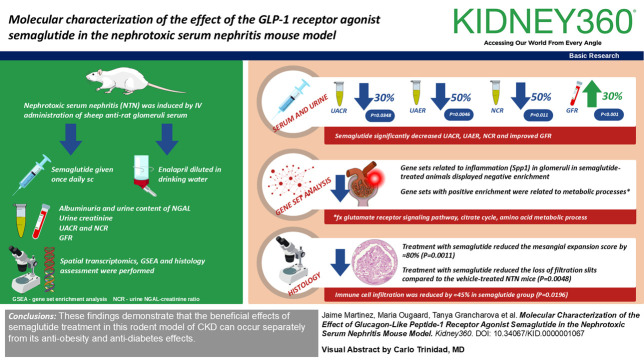

## Introduction

CKD is a public health concern due to its association with kidney failure, cardiovascular disease, and premature mortality and is estimated to affect half a billion people globally.^1,2^ Cardiovascular diseases, hypertension, diabetes, and obesity are major risk factors for development of CKD.^3–5^

Treatment with renin-angiotensin system inhibitors, sodium-glucose cotransporter 2 inhibitors, and a nonsteroidal mineralocorticoid receptor antagonist have been shown to improve kidney function and reduce risk of adverse cardiovascular outcomes in people with CKD.^6–12^ Interestingly, glucagon-like peptide-1 receptor agonists (GLP-1RA) have demonstrated promising effects on CKD. A review of seven cardiovascular outcome trials with GLP-1RA reported a beneficial 17% reduction in a composite kidney outcome index,^13^ and the trial Effect of Semaglutide versus Placebo on the Progression of Renal Impairment in Subjects With Type 2 Diabetes and CKD (FLOW) demonstrated treatment with semaglutide reduced the risk of clinically important kidney outcomes and death from cardiovascular causes.^14^ The underlying mechanisms are multifactorial and likely include improved glycemic control, reduced BP, reduced systemic inflammation, antiobesity effects, reduced oxidative stress and natriuresis as well as direct effects on renal blood flow, endothelial function, glomerular permeability, and renal inflammation.^14–17^ Other recent studies highlighted that the renal protective effects of GLP-1RA are not explained simply by improved glycemic control^18^ or reduction of body weight.^19^

In various mouse models, treatment with GLP1-RA improves kidney function and reduces inflammation and fibrosis.^20–22^ In animal models as well as humans, the GLP-1R is expressed only in vascular smooth muscle cells (VSMC) in the kidney,^23,24^ which suggests a role in regulation of renal blood flow. In various animal models, treatment with GLP1RA has in fact been reported to influence BP locally in the kidney,^25^ reduces hypertension and kidney pathology,^21^ and changes renal expression of genes in the renin-angiotensin-aldosterone system (RAAS).^22,23^ However, it is likely not the only mechanism of action, as another animal study reported treatment with liraglutide decreased expression of Ager, which correlated with improved kidney function.^26^ Effects of GLP-1RA on renal hemodynamics in humans vary between studies.^27^ A more complete understanding of effects of semaglutide treatment in the kidney in animal models of CKD would therefore be an important first step toward understanding effects in people with CKD.

The mouse nephrotoxic serum nephritis (NTN) model is a well-known CKD model, elicited by deposition of immune complexes in the glomerular basement membrane^28^ without hyperglycemia and obesity. The NTN model develops glomerulosclerosis, fibrosis, inflammation, tubular damage, elevated systemic markers of kidney damage, and albuminuria.^28^ The aim of this study was to characterize effects of semaglutide in the kidney of the NTN mouse model, to further clarify potential mechanisms of action in CKD, independent of effects on metabolic dysfunction. This was done by comprehensive analysis of kidney function parameters, gene expression effects, and histologic end points after treatment with semaglutide, with a special focus on the glomeruli (where pathologic alterations are initiated in the NTN model), areas in the kidney with VSMC (which express the GLP-1R) as well as areas with proximal tubules (PT).

## Methods

Detailed descriptions are available in the Supplemental Material and Supplemental Figures 5-7, 9, and 10.

### Animal Experiment

**Figure 1:**
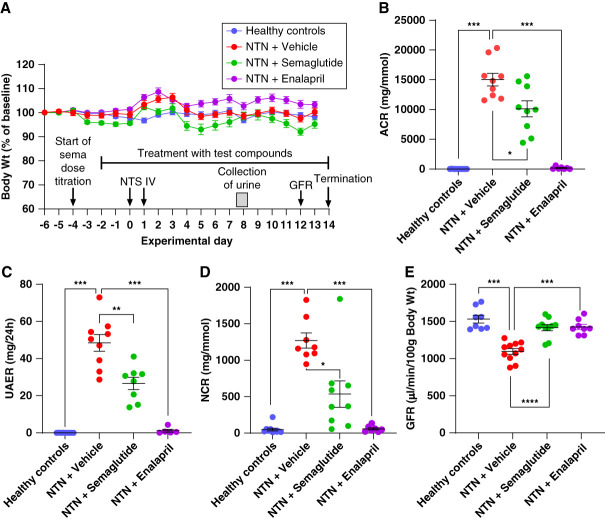
**Body weight and kidney function parameters.** (A) Combined timeline of the experiment and development in normalized body weight during the experimental period. (B) Urine ACR measured at day 7–8 (C) UAER at day 7–8. (D) Urinary NCR measured at days 7–8. (E) GFR measured at day 12. In all panels dots indicate observations from individual animals; number of animals in the different groups are listed in Supplemental Table 4. Horizontal lines indicate mean values±SEM. *, **, and *** indicate *P* < 0.05, 0.01 and 0.001, respectively, when groups were compared as shown. ACR, albumin-to-creatinine ratio; IV, intravenous; NCR, NGAL-to-creatinine ratio; NTN, nephrotoxic serum nephritis; NTS, nephrotoxic serum; UAER, Urinary albumin excretion rate.

Design of the animal experiment, performed according to National Institutes of Health (NIH) Guide for the Care and Use of Laboratory Animals, is presented in Figure 1A and Table 1. NTN was induced by intravenous (IV) administration of Sheep Anti-Rat Glomeruli Serum (nephrotoxic serum, cat. no. PTX-001S, Probetex Inc.). Semaglutide was administered by subcutaneous injection once daily, and enalapril was diluted in the drinking water. Urine was collected from all mice for 18 hours (day 7, 14:00 pm, to day 8, 8:00 am). GFR was measured on day 12 by preclinical transdermal GFR monitors (MediBeacon GmbH). After application of GFR, monitors mice were injected IV with 75 mg/kg FITC-sinistrin. The GFR monitor was removed after 1 hour, and GFR was subsequently calculated from the plasma t1/2 of FITC-sinistrin with the software MBStudio (MediBeacon GmbH). t1/2 of FITC-sinistrin is shown in Supplemental Figure 1. The animal experiment was terminated at day 14 after collection of blood samples. Right after euthanasia, both kidneys were collected and fixed in 10% neutral buffered formalin (VWR International).

**Table 1:** Design of animal experiment

Group and Disease Status)	NTN Induction	Number of Mice	Treatment
1 (Healthy controls)	No	12	None
2 (NTN)	Yes (IV d 0 and 1)	12	Vehicle
3 (NTN)	Yes (IV d 0 and 1)	12 (one animal euthanized before end of study)	Semaglutide 0.062 mg/kg 1× daily by SC injection starting d 2 (dose gradually increased at d -4 (0.021 mg/kg) and -3 (0.042 mg/kg)
4 (NTN)	Yes (IV d 0 and 1)	10 (one animal euthanized before end of study)	Enalapril 30 mg/kg, administered in drinking water from d 2

IV, intravenous; NTN, nephrotoxic serum nephritis; SC, subcutaneous.

Albuminuria and urine content of neutrophil gelatinase-associated lipocalin (NGAL) was assessed with ELISA (albumin: Bethyl Laboratories, cat.no. E90-134, NGAL: Bio-Techne, cat.no. NBP2-76750), while the creatinine urine level was assessed with a COBAS 6000 assay (Roche), all according to the manufacturer's instructions. Urinary albumin excretion rate (UAER) was calculated per 24 hours, and urine content of albumin and NGAL was normalized to the urine content of creatinine as the urine albumin-to-creatinine ratio (ACR) and urine NGAL-to-creatinine ratio (NCR).

Statistical analysis was performed with GraphPad Prism 10 (GraphPad Software) in a one-sided Brown-Forsythe ANOVA, followed by pairwise comparisons of groups in two-sided *t* tests with Dunnet correction. Adjusted *P* values < 0.05 were considered statistically significant.

### Single-Nucleus RNAseq

Single-nucleus RNAseq data from five mice (one healthy control, two vehicle-treated NTN animals, one semaglutide-treated animal, and one enalapril-treated animal) were generated with Chromium Fixed RNA kit (10× Genomics). Reads were mapped back to probes with CellRanger version 7.1.0 (10× Genomics) and analyzed using Seurat v5.1.

### Spatial Transcriptomics

Spatial transcriptomics (ST) data from kidney sections were generated with the Visium (10× Genomics) from 20 animals (healthy: No. =5, NTN+vehicle: No. =5, NTN+semaglutide: No. =5, NTN+enalapril: No. =5). Reads were processed with SpaceRanger version 1.3.1 or 2.1.1 (10× Genomics) and analyzed using Seurat v5.1.

### Differential Expression and Gene Set Enrichment Analysis

Differentially expressed genes in the ST data were explored by vehicle versus healthy, semaglutide versus vehicle, and enalapril versus vehicle using the Wilcoxon rank-sum test. Genes with a corrected *P* value < 0.05, an absolute log2 fold change >1, and expressed in ≥10% of the cell type population were considered statistically significant.

Gene set enrichment analysis (GSEA) based on all differentially expressed genes irrespective of fold change was conducted with clusterProfiler v.4.10.1^29^ by using the Kyoto Encyclopedia of Genes and Genomes, Reactome, and Gene Ontology terms databases as well as the Molecular Signature Database for identification of Hallmark gene sets. Pathways with a corrected *P* value < 0.05 were considered significant.

### Histology

Kidneys were processed for histology according to standard methods. For validation of ST results, *in situ* hybridization (ISH) was performed for *Ren1*, *Agtr1a*, and *Spp1*, and immunohistochemistry (IHC) was performed for the following proteins: CD45, kidney injury molecule 1 (KIM-1), aSMA, and collagen 3. ISH probes and primary antibodies used for IHC are listed in Table 2. Furthermore, filtration slit density in glomeruli was assessed, as described previously,^30^ and staining with Periodic Acid–Schiff (Sigma–Aldrich) was performed for semiquantitative scoring of mesangial expansion in 20 randomly selected glomeruli, as described previously.^28^ The fraction of the kidney cortex area and, for *Agtr1a* and *Spp1*, the glomerular area, with positive ISH, or IHC signals were quantified with the software Visiopharm or HALO (Indica). Statistical analysis was performed as described above for the other *in vivo* data.

**Table 2:** RNAscope probes used for *in situ* hybridization and primary antibodies used for immunohistochemistry

RNAscope Probes for ISH	Supplier and Catalog Number
Mm-*Ren1*	Bio-Techne, 433469-C1
Mm-*Agtr1a*	Bio-Techne, 404009-C1
Mm-*Spp1*	Bio-Techne, 435199-C1
Mm-*Glud1*	Bio-Techne, 538839-C1
Mm-*Ppib* (housekeeping gene)	Bio-Techne, 313919-C1
Mm-*Rho* (negative control)	Bio-Techne, 474809-C1
**Primary antibodies for IHC**	
Rabbit anti-CD45	Abcam, ab10558
Goat anti-mouse TIM-1/KIM-1/HAVCR1	R&D systems, AF1817
Rabbit anti-aSMA, clone EPR5368	Abcam, ab124964
Goat anti-type 3 collagen	Southern Biotech, 1330-01
Goat IgG (isotype control)	Jackson ImmunoResearch, 005-000-003
Rabbit IgG control (isotype control)	R&D systems, AB-105-C

HAVCR1, hepatitis A virus cellular receptor 1; IHC, immunohistochemistry; ISH, *in situ* hybridization; KIM-1, kidney injury molecule 1.

## Results

### Treatment with Semaglutide Did not Influence Body Weight and Improved Kidney Function

Development in body weight followed a comparable pattern in all groups (Figure 1A). Treatment with semaglutide had a beneficial effect on parameters of kidney function and significantly reduced urine ACR by ≈30% (*P* = 0.0348, Figure 1B), UAER by ≈50% (*P* = 0.0046, Figure 1C) and lowered urine NCR by ≈50% (*P* = 0.011, Figure 1D). GFR was decreased in vehicle-treated NTN mice compared with healthy mice, and treatment with semaglutide increased GFR significantly by ≈30% compared with vehicle-treated NTN mice (*P* < 0.001, Figure 1E). Treatment with enalapril essentially normalized urine ACR, UAER, NCR, and GFR to levels comparable with those observed in healthy mice (Figure 1, B–E).

### ST Combined with snRNAseq Allowed Identification of Relevant Kidney Compartments

**Figure 2:**
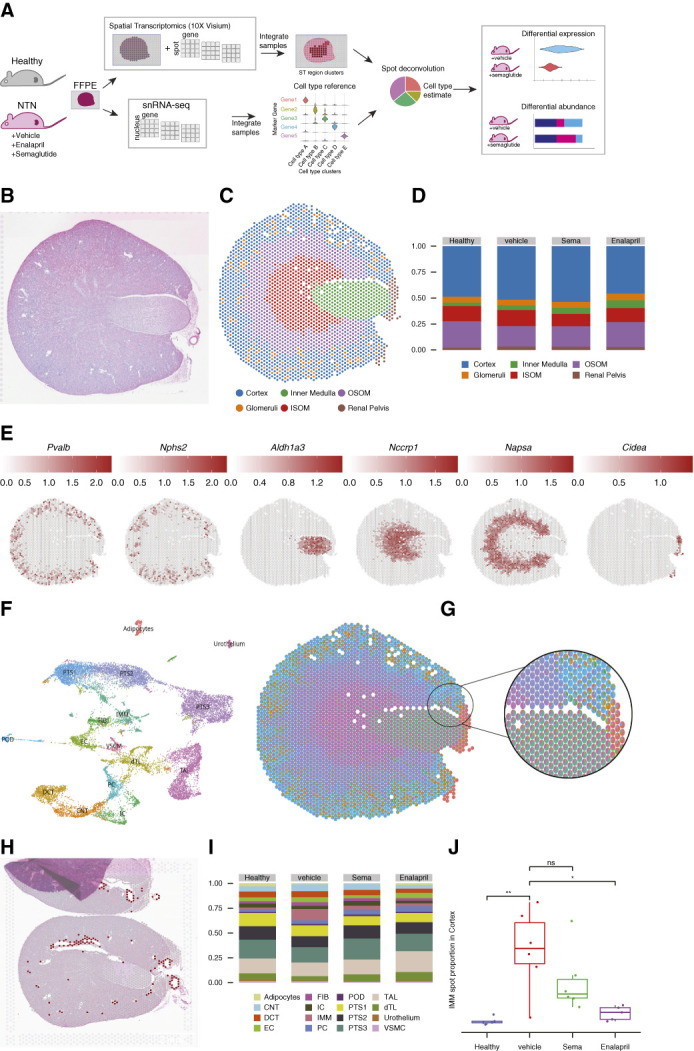
**ST and snRNAseq allowed identification of kidney compartments and cell types.** (A) *In silico* workflow for ST and snRNAseq. (B) HE-stained image of kidney section used for ST. (C) Annotation of ST spots to the major kidney compartments. (D) Relative proportions ST spots in the major kidney compartments across treatment groups. (E) Spatially variable genes as markers of major kidney compartments. (F) Annotated snRNAseq data. (G) With the snRNAseq data ST data were deconvoluted, so proportions of cell types within each ST spot could be calculated. (H) With the deconvoluted ST data, spots with high proportions of cell types of interest could be identified, for example, spots with high proportions of SMC. (I) Proportions of all cell types identified in the kidney across treatment groups. (J) Proportion of immune cells in the treatment groups in the cortex. Dots indicate observations from individual animals, while horizontal lines indicate the median or quartiles of the data distribution; * and ** indicate *P* < 0.05 and 0.01, respectively, in the shown comparisons. CNT, connecting tubule; DCT, distal convoluted tubule; EC, endothelial cell; dTL, ascending thin limb cells; FIB, fibroblasts; HE, hematoxylin and eosin; IC, intercalated cell; IMM, immune cells; ISOM, inner stripe of outer medulla; OSOM, outer stripe of outer medulla; PC, principal cells; POD, podocytes; SMC, smooth muscle cells; ST, spatial transcriptomics; TAL, thick ascending limb cells; VSMC, vascular smooth muscle cells.

ST and snRNAseq data obtained from the same mice were used to investigate the effect of different treatments on the molecular landscape of kidney tissue (Figure 2A). Delineating the major anatomical compartments in mouse kidney solely through manual visual inspection of images is time-consuming (Figure 2B). Unsupervised clustering of the spot-based ST data followed by manual refinement required less time and allowed for more precise identification of the renal compartments (Figure 2C). The relative proportions of kidney compartments were comparable across the treatment groups (Figure 2D). Through the identification of these kidney compartments, spatially variable genes that are uniquely expressed in specific regions were identified (such as *Pvalb* for the cortex [Figure 2E]). These genes are valuable markers for kidney compartments also in future studies.

Kidney cell types were identified in the snRNAseq data (Figure 2F) and used for deconvoluting the Visium ST data. Cell type proportions for each spot (Figure 2G) were then used to select spots in the kidney tissue with a high proportion of cells of interest, such as VSMC (Figure 2H; kidney VSMC express GLP-1R) and PT cells (previous studies reported effects of GLP-1RA on natriuresis through effects on PT cells). The deconvoluted Visium data also allowed us to monitor if cell type proportions varied across disease states. For example, the immune cell proportion increased in vehicle-treated NTN animals and was reduced after treatment with semaglutide (Figure 2, I and J).

### Treatment with Semaglutide or Enalapril Influenced Multiple Cellular Functions

**Figure 3:**
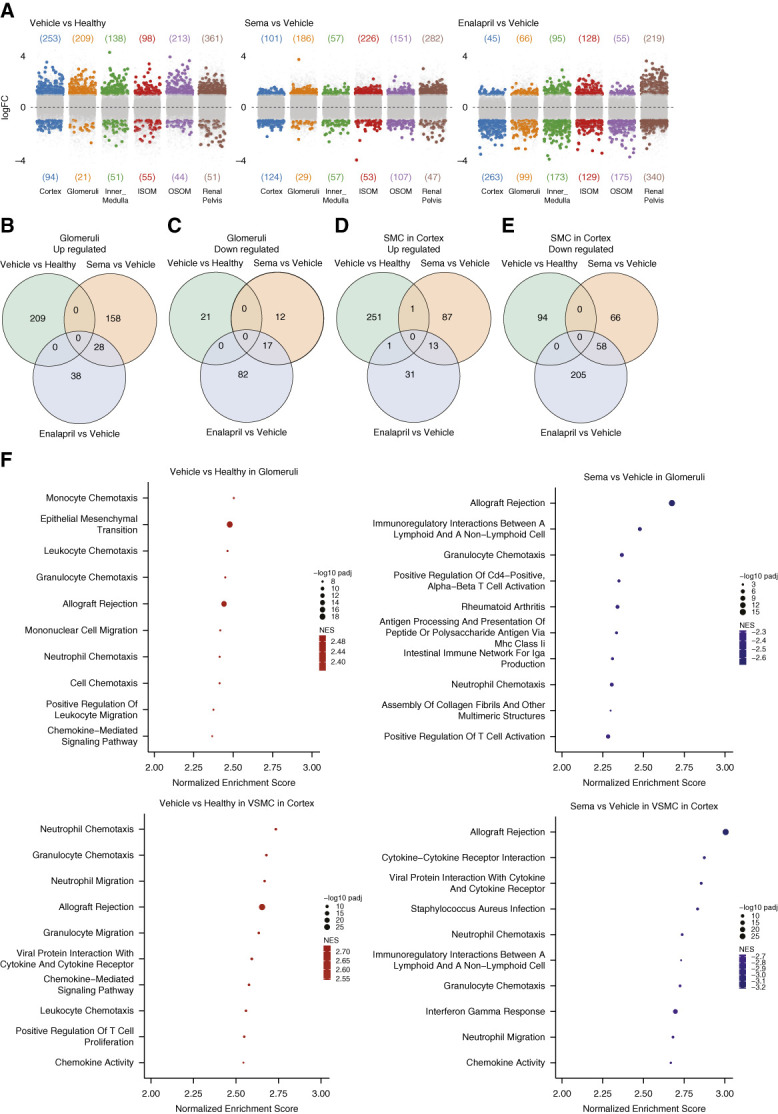
**Differentially expressed genes and GSEA.** (A) Numbers of differentially expressed genes for vehicle-treated NTN mice relative to healthy, semaglutide-treated animals relative to vehicle-treated, and enalapril-treated animals relative to vehicle-treated, in the kidney compartments cortex, glomeruli, OSOM, ISOM, inner medulla, and renal pelvis. (B) Venn diagram for genes upregulated in glomeruli, when vehicle-treated NTN mice, semaglutide-treated NTN mice, and enalapril-treated NTN mice were compared with their corresponding control groups. (C) Venn diagram for genes downregulated in glomeruli, when vehicle-treated NTN mice, semaglutide-treated NTN mice, and enalapril-treated NTN mice were compared with their corresponding control groups. (D) Venn diagram of genes upregulated in VSMC, when vehicle-treated NTN mice, semaglutide-treated NTN mice, and enalapril-treated NTN mice were compared with their corresponding control groups. (E) Venn diagram of genes downregulated in VSMC, when vehicle-treated NTN mice, semaglutide-treated NTN mice, and enalapril-treated NTN mice were compared with their corresponding control groups. (F) Top 10 significant enriched gene sets. Comparisons and kidney compartment are described on the figure. FC, fold change; GSEA, Gene set enrichment analysis; SMC, smooth muscle cells.

The numbers of differentially expressed genes in kidney compartments are shown in Figure 3A, and all differentially expressed genes are listed in Supplemental Table 1. Venn diagrams for upregulated and downregulated genes are shown in Figure 3, B–E (glomeruli and VSMC) and Supplemental Figure 2 (PT). In the glomeruli, there was very limited overlap only between the semaglutide-treated and enalapril-treated groups (Figure 3, B and C). In the VSMC compartment, only one gene overlapped between vehicle-treated mice and mice treated with semaglutide or enalapril, while more common genes were observed between the two latter groups (Figure 3, D and E). A comparable pattern with no common genes between vehicle-treated mice and mice treated with semaglutide or enalapril was observed in the PT (Supplemental Figure 2).

The top 10 enriched gene sets are shown in Figure 3F for vehicle-treated versus healthy, and semaglutide versus vehicle-treated animals, in glomeruli and VSMC. All enriched gene sets are shown for all comparisons in Supplemental Table 2. In glomeruli, the vehicle-treated NTN mice displayed upregulation of genes involved in multiple immune functions, such as allograft rejection, complement activation, and immunoglobulin-mediated immune response. Furthermore, multiple enriched genes are involved in fibrogenesis, such as collagen formation, extracellular matrix organization, and epithelial mesenchymal transition. Of note, the genes *Bgn*, *Dcn*, *Fn1*, *Col4a1*, *Lama2*, and *Nid1*, which encode glycoproteins or proteins in the mesangial matrix,^31^ were all significantly upregulated (Supplemental Table 3). Interestingly, gene sets involved in carboxylic acid metabolism and gene sets involved in oxidative phosphorylation had a negative enrichment score in glomeruli from NTN kidneys compared with healthy mice, in agreement with previous studies.^32,33^

Gene sets enriched in glomeruli in semaglutide-treated animals relative to vehicle-treated most frequently displayed a negative enrichment score. These enriched gene sets represented mainly inflammatory processes, and few biologic functions were related to fibrogenesis (*e.g*., assembly of collagen fibrils). These biologic functions were often positively enriched in glomeruli in vehicle-treated NTN mice. The few gene sets with positive enrichment were related to metabolic processes (*e.g*., glutamate receptor signaling pathway, citrate cycle, amino acid metabolic process; Supplemental Table 2), which indicate that treatment with semaglutide could influence immunometabolism. Interestingly, the regulation of systemic arterial BP mediated by a chemical signal function was among the top enriched pathways. This gene set included *Ren1*.

In enalapril-treated animals, negatively enriched gene sets were also related to inflammation and fibrosis, while many positively enriched gene sets were related to metabolic functions (Supplemental Figure 3). As expected, the Regulation of systemic arterial BP by renin-angiotensin function was among the positively enriched gene sets.

GSEA in the VSMC compartment generally followed the same trends as in glomeruli, *i.e*., in the vehicle-treated NTN mice immune functions and processes involved in fibrosis displayed positive enrichment, while oxidative phosphorylation processes were negatively enriched. This pattern was generally reversed in both semaglutide-treated and the enalapril-treated animals. Especially gene sets covering various functions within amino acid metabolism were positively enriched in the semaglutide-treated or enalapril-treated groups. In the enalapril-treated animals, several gene sets comprising genes in the RAAS also displayed positive enrichment (Supplemental Table 2).

GSEA in PT appeared fully comparable with the pattern in glomeruli and areas with VSMC (see Supplementary Results).

### Histologic Analysis Validated Findings from ST and Revealed Beneficial Effects of Semaglutide on Histopathologic Parameters

To validate findings from ST, ISH was performed for *Ren1* and *Agtr1a* (representing the RAAS) and *Spp1* (representing immune function and tissue remodeling). Furthermore, IHC was performed for CD45 (general immune cell marker), KIM-1 (injury marker in tubular epithelium), aSMA and collagen 3 (markers of fibrogenesis and fibrosis), and the mesangial expansion was scored semiquantitatively. The mean fold changes for the experimental interventions for the selected genes assessed with Visium and ISH or IHC are listed in Supplemental Table 3. We observed good agreement for most of the comparisons, with treatment effects in the same direction.

**Figure 4:**
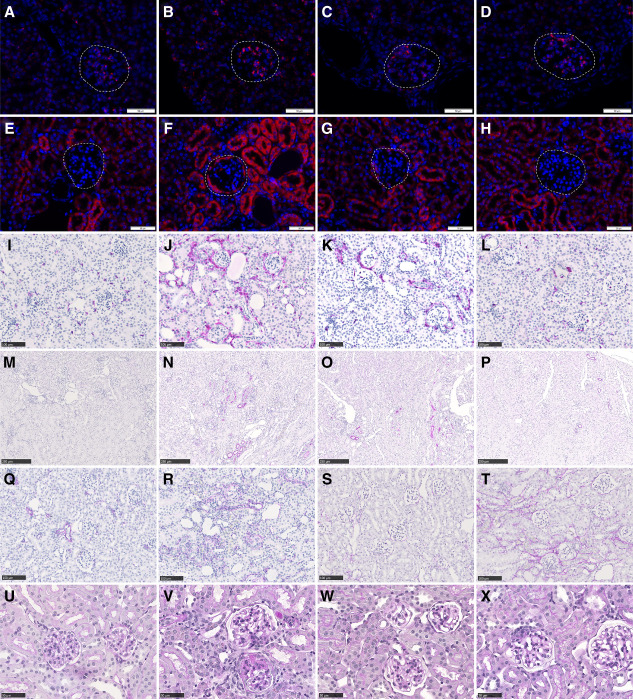
**Representative histologic images.** *Agtr1a* mRNA (red signal) in a healthy control mouse (A), a vehicle-treated NTN mouse (B), a semaglutide-treated NTN mouse (C), and an enalapril-treated NTN mouse (D). Glomeruli are outlined with dashed lines. *Spp1* mRNA (red signal) in a healthy control mouse (E), a vehicle-treated NTN mouse (F), a semaglutide-treated NTN mouse (G), and an enalapril-treated NTN mouse (H). Glomeruli are outlined with dashed lines. Note how *Spp1* expression increases in vehicle-treated NTN mouse compared with healthy, also in parietal cells lining the Bowmans capsule (F). CD45 staining (purple signal) in a healthy control mouse (I), a vehicle-treated NTN mouse (J), a semaglutide-treated NTN mouse (K), and an enalapril-treated NTN mouse (L). Note how the amount of CD45-positive cells increases in the interstitium between tubuli and around glomeruli. KIM-1 staining (purple signal) in a healthy control mouse (M), a vehicle-treated NTN mouse (N), a semaglutide-treated NTN mouse (O), and an enalapril-treated NTN mouse (P). As expected, KIM-1 staining is virtually absent in healthy control mice (M). Alpha-SMA staining (purple signal) in a healthy control mouse (Q) and a vehicle-treated NTN mouse (R). Collagen 3 staining (purple signal) in a healthy control mouse (S) and a vehicle-treated NTN mouse (T). Note the increased amount of aSMA as well as collagen 3 in the interstitium in vehicle-treated NTN mice, reflecting development of fibrosis (R and T, respectively). PAS staining in a healthy control mouse (U), a vehicle-treated NTN mouse (V), a semaglutide-treated NTN mouse (W), and an enalapril-treated NTN mouse (X). Note the clear mesangial expansion in the vehicle-treated NTN mouse (V). PAS, Periodic Acid-Schiff.

**Figure 5:**
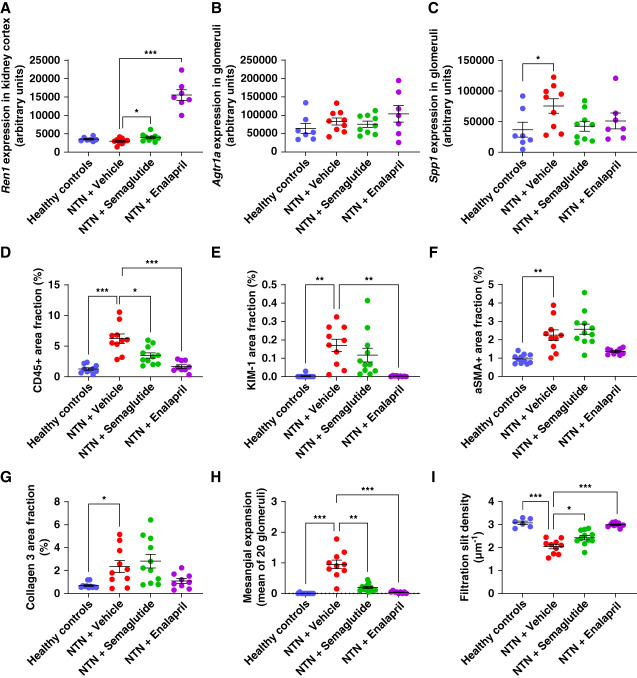
**Quantification of histology end points by image analysis.** (A) Ren1 mRNA expression in kidney cortex. (B) *Agtr1a* mRNA expression in glomeruli. (C) *Spp1* mRNA expression in glomeruli. (D) CD45 expression in kidney cortex. (E) KIM-1 expression in kidney cortex. (F) aSMA expression in kidney cortex. (G) Collagen 3 expression in kidney cortex. (H) Mean mesangial expansion score. (I) Filtration slit density in glomeruli. In all panels, symbols indicate observations from individual animals, and horizontal lines indicate mean values±SEM. *, **, and *** indicate *P* < 0.05, 0.01, and 0.001, respectively, when groups were compared as indicated on the panels.

Representative histologic images of ISH for *Agtr1a* and *Spp1* and IHC for CD45, KIM-1, aSMA, and collagen 3 as well as Periodic Acid–Schiff-staining are shown in Figure 4, and image analysis results are shown in Figure 5. Representative images of podocin staining for assessment of filtration slit density is shown in Supplemental Figure 4. *Ren1* expression was significantly increased in semaglutide and enalapril-treated animals (Figure 5A) in agreement with ST results. *Agtr1a* mRNA expression was observed mainly in glomeruli and did not vary significantly between treatment groups (Figure 4, A–D and Figure 5B). *Spp1* mRNA expression was seen widespread across kidney compartments and increased significantly also in glomeruli in vehicle-treated NTN mice compared with healthy (*P* = 0.0303, Figure 4, E–H and Figure 5C). The average glomerular *Spp1* signal was decreased ≈40% in animals treated with semaglutide or enalapril, although this did not reach statistical significance.

Immune cell infiltration, identified by CD45^−^staining, increased markedly in the kidney interstitium between tubuli and around glomeruli in NTN-mice and was reduced by ≈45% in the semaglutide-treated group (*P* = 0.0196, Figure 4, I–L and Figure 5D). Induction of disease also resulted in a pronounced increase in KIM-1 staining (Figure 4, M–P and Figure 5E), and we observed a nonsignificant trend that KIM-1 staining decreased in semaglutide-treated animals by ≈40%. Vehicle-treated NTN mice also displayed increased amounts of aSMA (Figure 4, Q and R and Figure 5F) and collagen 3 (Figure 4, S and T and Figure 5G), which reflects fibrosis development. However, in this 14-day experiment treatment with semaglutide or enalapril had no significant impact on protein levels of these fibrosis end points. In the glomeruli, mesangial expansion was significantly increased on induction of disease (Figure 4, U–X and Figure 5H) and treatment with semaglutide reduced the mesangial expansion score by ≈80% (*P* = 0.0011, Figure 5H). Furthermore, treatment with semaglutide reduced the loss of filtration slits compared with the vehicle-treated NTN mice (*P* = 0.0048, Figure 5I). Treatment with enalapril resulted in more pronounced beneficial effects on kidney inflammation, tubular damage, mesangial expansion, and filtration slit density (Figure 5, D–I).

## Discussion

The main finding in this study is that treatment with semaglutide improved kidney function as well as histopathologic parameters in the kidney in the NTN model. It is the first study with semaglutide in a normoglycemic and nonobese model of CKD, and the beneficial effects of semaglutide on kidney function clearly occurred independent of antidiabetic and antiobesity effects. This is in agreement with previous indications from people with CKD.^18,19^ Exploration of the underlying changes in gene expression revealed treatment with semaglutide exerted anti-inflammatory effects, and somewhat more subtle, antifibrotic effects in glomeruli and areas with VSMC.

Previous studies in rodent models indicate that the beneficial effects of GLP1-RA on kidney parameters are mediated through effects on kidney blood flow. For example, GLP-1RA resulted in vasodilation of the afferent arterioles, increased renal blood flow, and GFR.^25,34^ A vasodilatory effect could result from direct effect of GLP-1 on the VSMC where GLP-1R is expressed^24^ and through effects on the RAAS. It was previously observed that treatment of rats with GLP-1RA increased expression and/or activity of angiotensin II converting enzyme^35–38^ and resulted in increased plasma concentrations of angiotensin1,7.^36^ In the NTN model, the expression of Mas1, the receptor for angiotensin1,7, was previously increased in glomeruli from liraglutide-treated animals,^22^ which indicate that the beneficial effects of GLP1-RA in the NTN model could be mediated by increased angiotensin1,7-Mas1 signaling. Increased expression of renin in the kidney after treatment with GLP-1RA^22,23^ could be a compensatory mechanism on increased metabolism of angiotensin II and/or a response to vasodilation of afferent arterioles. We also observed increased expression of *Ren1* after treatment with semaglutide but did not detect significant effects on other genes in the RAAS (see also Supplementary Results and Supplemental Figure 8). This is an important novel finding, and future studies are needed to clarify how treatment with GLP-1R agonists might influence the RAAS at protein level as well as kidney blood flow in general.

In humans, it is less clear how treatment with GLP-1RA influence kidney blood flow and the RAAS. The results vary between studies and have been suggested to depend on study population and/or the applied doses of GLP-1.^27^ In healthy men, acute treatment resulted in no change GFR or renal plasma flow (RPF),^39–41^ while GFR and RPF were increased after treatment in obese men,^42^ which could indicate dilation of afferent arterioles. A study in people with type 2 diabetes in contrast reported increased resistance in the afferent arterioles, which, however, did not decrease the GFR or RPF.^43^ In trials of longer duration, GFR declined slower in participants treated with semaglutide or dulaglutide compared with the relevant control groups.^14,44^ Several of the studies mentioned above also explored if the RAAS was affected by treatment with GLP-1RA. Some studies observed no effect on plasma renin levels,^41,45,46^ while another study reported decreased levels of plasma renin.^40^ Interestingly, acute treatment of healthy men with liraglutide was found to decrease plasma angiotensin II levels with 20%,^39,41^ and a similar observation was seen after acute treatment of patients with type 2 diabetes.^46^ However, no change in plasma levels of angiotensin II was seen after prolonged treatment of type 2 diabetic people with GLP-1RA.^47,48^ Future studies are therefore needed to clarify if effects on kidney blood flow and the RAAS are linked to the beneficial effect of GLP-1RA in human CKD patients. The trial Renal Mode of Action of Semaglutide in Patients With Type 2 Diabetes and CKD aims at elucidating the mechanisms behind the beneficial effects of semaglutide in human CKD patients, and several of the end points are related to kidney blood flow and resulting oxygenation in the kidney tissue.

This study revealed effects which has not been described previously in the NTN model. *Spp1* expression increased in NTN kidneys in tubular epithelium as well as in parietal epithelial cells in the glomeruli, in good agreement with previous findings in other animal models of CKD and human patients.^49,50^ Treatment with semaglutide or enalapril interestingly reduced expression of *Spp1*. *Spp1* functions to maintain tubular mineral solubility but has also been reported to promote differentiation of fibroblasts to myofibroblasts and thereby contribute to fibrosis progression.^51^ Furthermore, this finding is also linked to effects on inflammation, as it is known that a subset of macrophages in CKD express *Spp1* and that these interact with damaged cells and have a profibrotic action.

In this study, treatment with semaglutide had a prominent anti-inflammatory effect, comparable with effects of GLP1-RA in other studies and animal models.^21,22,26^ The present results from the NTN model demonstrate that anti-inflammatory effects of semaglutide cannot solely be indirect effects mediated by normalization of whole-body metabolism. Although our data do not unequivocally point to reduction of inflammation as a causative mechanism for the renal functional improvement following treatment with semaglutide, previously reported preclinical and clinical evidence strongly suggest that both systemic and locally acting anti-inflammatory effects contribute to the clinical benefits seen with semaglutide in CKD and other chronic diseases.^52^

A recent study with a diabetic as well as a normoglycemic animal model of CKD interestingly observed decreased expression of *Ager* after treatment with liraglutide, and this correlated with improved kidney function.^26^ However, in this study, no *Ager* expression was observed in the vehicle-treated animals, and in healthy animals as well as semaglutide-treated and enalapril-treated animals, *Ager* expression was at minimal levels (data not shown).

The effects of semaglutide on several end points appeared somewhat weak in this study. One could speculate that this was a result of a relatively small window for pathologic alterations in this 14-day study with the NTN model. For example, the mean mesangial expansion score and KIM-1 area fractions were considerably lower in our study than in another study with a pathologically more progressed model.^21^ It could potentially be easier to detect treatment effects in a more diseased model. The NTN model must also be seen as a model of the acute and subchronic stages of CKD, and although it develops fibrosis, the model does not show progressive loss of GFR and worsening of urine albumin-to-creatinine ratio over time. This study was also limited to comprehensive analysis of the effects of semaglutide in the kidney. There might be important effects on systemic circulation and components in the RAAS outside the kidney. Finally, while we could validate many of the findings from analysis of ST data with ISH and IHC, it is important to note that analysis of the ST data was performed at the spot level, while analysis of ISH/IHC data was based on area fractions determined per section, which will contribute to differences in results.

In conclusion, treatment with semaglutide improves kidney function and pathology in the mouse NTN model. Transcriptomics data demonstrated that semaglutide had pronounced anti-inflammatory and antifibrotic effects and indicated beneficial effects on renal hemodynamics. These results in the NTN model show that beneficial effects of semaglutide can occur independent of metabolic effects.

## Supplementary Material

## Data Availability

Original data generated for the study are or will be made available in a public access repository upon publication. Data Type: Aggregated Data. Repository Name: Other. The code for this study is available at https://github.com/Jaimomar99/GLP-1RA_NTNmodel. All data included in this manuscript have been deposited in BioStudies, accession number S-BSST2222.

## Declarative Statements

All animal experiments were conducted in accordance with the NIH Guide for the Care and Use of Laboratory Animals or an equivalent standard that meets or exceeds the ethical and welfare requirements outlined in the NIH Guide. All protocols were approved by the appropriate institutional animal care and use committee.

## Supplemental Material

Supplemental Materials and Methods.

Supplemental Results.

Supplemental Table 1. Differentially expressed genes genes identified in the ST data.

Supplemental Table 2. GSEA of the ST data.

Supplemental Table 3. Comparison of relative differences between groups (fold changes) observed in Visium ST data to relative differences observed with ISH or IHC.

Supplemental Table 4. Number of animals per group included in ISH and IHC assays.

Supplemental Table 5. Abbreviations.

Supplemental Figure 1. t1/2 of FITC-sinistrin. Symbols are observations from individual animals; horizontal lines and error bars indicate mean ± SEM. *** indicate P.

Supplemental Figure 2. Venn diagram of genes regulated in PT, when vehicle-treated NTN mice, semaglutide-treated NTN mice, and enalapril-treated NTN mice were compared with their corresponding control groups. A: Upregulated genes. B: Downregulated genes.

Supplemental Figure 3. Top 10 significant enriched gene sets in Enalapril versus vehicle. Compartments are described on the figure.

Supplemental Figure 4. Representative images of podocin staining.

Supplemental Figure 5. Uniform manifold approximation and projection of the snRNAseq data colored by treatment.

Supplemental Figure 6. Key markers showing cell type annotation in the snRNAseq dataset.

Supplemental Figure 7. Uniform manifold approximation and projection of the ST dataset colored by treatment.

Supplemental Figure 8. Expression levels of key genes belonging to the RAAS in the different kidney compartments for each experimental group.

Supplemental Figure 9. Relative abundance of kidney compartments in each individual animal.

Supplemental Figure 10. Relative abundance of cell types in each individual animal.

Supplemental References.
