# Supplementary material for: Molecular Characterization of the Effect of Glucagon-Like Peptide-1 Receptor Agonist Semaglutide in the Nephrotoxic Serum Nephritis Mouse Model
Source: Kidney360. 2025 Dec 3;7(4):728–40. doi: 10.34067/KID.0000001067 (PMC13134791; doi:10.34067/KID.0000001067)
Supplement: Supplementary file 2 [file kidney360-7-728-s002.pdf]

Supplemental Material contents

Supplemental Materials and Methods
Supplemental Results
Supplemental Table 1
Supplemental Table 2
Supplemental Table 3
Supplemental Table 4
Supplemental Figure 1
Supplemental Figure 2
Supplemental Figure 3
Supplemental Figure 4
Supplemental Figure 5
Supplemental Figure 6
Supplemental Figure 7
Supplemental Figure 8
Supplemental Figure 9
Supplemental Figure 10
Supplemental References

Supplemental Materials and Methods

Design of animal experiment

All work with laboratory animals in this study was performed under a license granted by National Danish authority the Animal Experiments Inspectorate and in accordance with NIH Guide for the Care and Use of Laboratory Animals. Fifty female CD1 mice (Charles River, Germany) were purchased at 8-10 weeks of age and acclimatized for a week before experimental procedures were initiated. The mice were housed 10 mice per cage in Macrolone type IV cages, with unrestricted access to a standard rodent chow diet (Altromin 1234, Brogaarden, Denmark), tap water (non-chlorinated, non-acidified) and with a 12:12 hour light-dark cycle. The design of the animal experiment is shown in Table 1 and Figure 1A. Nephrotoxic serum nephritis (NTN) was induced in mice in group 2, 3 and 4 by iv administration of Sheep Anti-Rat Glomeruli Serum (nephrotoxic serum (NTS), cat. no. PTX-001S, San Antonio, USA) containing sheep anti-rat antibodies directed against the glomerular basement membrane) on days 0 and 1 (daily dose: 100 µl 80% NTS). Mice were treated with either vehicle, semaglutide (Novo Nordisk A/S) or enalapril (Sigma-Aldrich). Semaglutide was administered by sc injection once daily, formulated in a vehicle containing 0.007

% polysorbate 20, 50 mM phosphate and 70 mM NaCl, at pH 7.4. The dose of semaglutide was gradually increased starting at day -4 to the final daily dose of 0.062 mg/kg. Enalapril was diluted in the drinking water to a concentration of 0.18 mg/ml, and the estimated daily dose was 30 mg/kg. The animal experiment was terminated at day 14. At termination the mice were anaesthetised in isoflurane for collection of a terminal blood sample from the heart in EDTA-coated tubes (Sarstedt). Right after euthanasia, both kidneys were collected, cut in half with a transverse section and fixed in 10 % neutral buffer formalin (VWR International).

Collection of urine

For collection of urine, all mice were placed in metabolic cages with free access to water and powdered standard chow for 18 hours on day 7, from 14:00 PM until day 8 8:00 AM. The urine volume was quantified and stored at -20°C until further analysis.

Quantification of the glomerular filtration rate

Glomerular filtration rate (GFR) was measured on day 12 by preclinical transdermal GFR monitors (MediBeacon GmbH, Mannheim, Germany). In short, a square of 2x2 cm on the back of the mice were depilated 24 hours prior to GFR measurements. The mice were then lightly anaesthetised, the GFR monitor was adhered to the depilated area by adhesive tape, and the mice were injected iv (tail vein) with 75 mg/kg FITC-sinistrin. Animals were conscious and free to move around in their cage during the GFR measurement. The GFR monitor was removed after one hour and GFR was subsequently calculated from the plasma half-life of FITC-sinistrin with the software MBStudio (MediBeacon GmbH) according to the manufacturer's instructions.

Statistical analysis of in vivo data

Statistical analysis was performed with the software GraphPad Prism 10 (GraphPad Software, Boston, MA, US). All in vivo data were analysed in a one-sided Brown-Forsythe ANOVA, followed by pairwise comparison of each group to the vehicle-treated NTN group, with Dunnets correction for multiple parallel comparisons. Adjusted p-values <0.05 were considered statistically significant.

snRNAseq analysis

Nuclei from FFPE kidney samples from five mice (one healthy control, two vehicle-treated NTN animals, one semaglutide treated animal and one enalapril-treated animal) were isolated from 25 µm thick sections, according to protocols from 10x Genomics. Single-nucleus RNA-seq data were generated with Chromium Fixed RNA Profiling Reagent Kits, containing the Chromium Mouse Transcriptome Probe Set v1.0.1, and a Chromium iX instrument (all from 10x Genomics), according to the manufacturer's instructions. In total, 20,000 nuclei were profiled (≈4,000 nuclei from each animal). Libraries were sequenced on a NovaSeq 6000 instrument (Illumina), according to the manufacturer's instructions with ≈10,000 paired-end reads per nuclei. Reads were mapped back to probes in individual cells with cell ranger version 7.1.0 (10x Genomics).

The matrices output from cell ranger were read using Seurat v5.1 for downstream analysis. Quality control was performed by retaining cells with gene number > 200, UMI number > 500 and mitochondrial percentage < 30. In addition, doublets were removed using the Scrublet algorithm, removing cells with a score higher than 0.3[1]. Samples were merged and normalized using SCTransform with v2 regularization[2]. Subsequently, the samples were integrated using Harmony[3] to correct by batch group and diagnosis (Supplemental Figure 5). The integration embedding was used for dimensionality reduction and clustering at different resolutions. Silhouette score was used to obtain the optimal number of clusters, and clusters were annotated with cell types using well-known markers from the literature[4,5]. To confirm cell types, genes enriched in each cluster (cluster vs rest) were checked in PanglaoDB[6] and Enrichr[7] (Supplemental Figure 6).

Spatial transcriptomics

ST data were generated with the Visium platform (10x Genomics) from FFPE kidney sections. In a first round, 14 samples were analyzed (healthy: n=3, NTN+Vehicle: n=3, NTN+semaglutide: n=4, NTN+enalapril: n=4) with the Visium for FFPE v1 assay and Visium Mouse Transcriptome Probe Set v1.0 (all from 10x Genomics). In a subsequent round, 6 more samples were analyzed (healthy: n=2, NTN+Vehicle: n=2, NTN+semaglutide: n=1, NTN+enalapril: n=1) with the Visium for FFPE v2 assay and Visium Mouse Transcriptome Probe Set v1.0 (10x Genomics). All procedures were performed according to protocols provided by the manufacturer. Libraries were sequenced on a NovaSeq 6000 instrument (Illumina), according to the manufacturer's instructions. Reads were mapped back to probes and spots as well as aligned to the corresponding histology image with

space ranger version 1.3.1 (round 1) or space ranger version 2.1.1 (round 2). The output data from Space Ranger was processed using the *Load10X_Spatial* function from the Seurat package. Each sample underwent individual quality control, wherein low-quality spots and genes were filtered out based on quantile considerations. Normalization, integration, dimensionality reduction, and clustering were performed following the same approach as the snRNAseq dataset (Supplemental Figure 7).

For the annotation of distinct kidney regions, clusters were compared to identify markers enriched in each region. Additionally, the clusters were mapped back to individual images to assess region overlap within the kidney. Six regions were delineated: Cortex, Outer Stripe of the Outer Medulla (OSOM), Inner Stripe of the Outer Medulla (ISOM), Glomeruli, Inner Medulla, and Renal Pelvis. Furthermore, to validate the identification of glomeruli, pathologists manually annotated the spots in the LoupeBrowser software and compared these areas with the clusters. The relative abundances of kidney compartments in each individual animal are shown in Supplemental Figure 9.

The spatial integrated object underwent deconvolution using the Bayesian model Cell2location[8] with the snRNAseq serving as reference, as described previously[9] . The snRNAseq model was trained over 500 epochs. Due to the size of the spatial dataset and limits in GPU memory, it was partitioned into three random subsets while ensuring similarity in sample and diagnosis across the subsets. The three regression models were trained for 20,000 epochs considering that within each spot 8 cells were included.

Differential Expression Analysis

Differential expression analysis was conducted at the regional level to investigate changing molecular mechanisms within the different treatments in the ST dataset. The entire transcriptome was evaluated across three distinct comparisons: vehicle vs. healthy, semaglutide vs. vehicle, and enalapril vs. vehicle. Genes with a p-value below 0.05 after multiple-testing correction, an absolute log2 fold change greater than 1, and expressed in at least 10% of the cell type population were considered statistically significant. This analysis was performed using the function FindAllMarkers from Seurat, employing a non-parametric test, specifically the Wilcoxon rank sum test.

Gene set enrichment analysis

A functional analysis was conducted across the three comparisons utilizing clusterProfiler v.4.10.1[10] and encompassed three databases: Kyoto Encyclopedia of Genes and Genomes (KEGG), Reactome and Gene Ontology terms (GO). Additionally, the Molecular Signature Database (MSigDB) was used to investigate the Hallmarks gene sets. Only pathways with a p-value below 0.05 after multiple-testing correction were investigated. For visualization, pathways were arranged by the absolute value of the Normalized Enrichment Score, and the top 10 pathways were plotted.

Cell type composition

The cell type composition across the four different treatments was assessed using snRNAseq data, while region composition was examined using ST data. Furthermore, the integration of these datasets allowed for the deconvolution of spot cell type proportions, enabling an investigation of cell type composition per treatment and region. Relative abundances of cell types in each individual animal is shown in Supplemental Figure 10.

The combination of snRNAseq and ST facilitated the identification of spatially distinct regions with high cell type abundance. By leveraging the integration of snRNAseq and ST, specific spots enriched with vascular smooth muscle cells (VSMC) in the cortex and spots enriched with proximal tubular (PT) cells in the cortex, glomeruli, and OSOM regions were selected for further detailed analysis. To assess the statistical significance of these proportions, pairwise comparisons were conducted using the Wilcoxon rank sum test via the ggpubr package v.0.6.0[11].

Kidney histology

Following fixation in 10% neutral buffered formalin 48 hours, kidneys were dehydrated and embedded in paraffin. The right kidney from each animal was used for analysis of filtration slit density, performed at the CRO NIPOKA GmbH as described below and in a recent publication[12]. The left kidney from each animal was used for in situ hybridization, immunohistochemistry and histochemistry.

In situ hybridization for the genes Ren1, Agtr1a, Spp1, the houskeeping genes Ppib and the negative control gene Rho was performed with the commercially available RNAscope probes listed in table 2 (all from Bio-Techne) in single-plex assays on kidney sections of 4.5 µm

thickness essentially as described previously[13], except that Cy5 (red flourophore) was used for detection of the mRNA signal and nuclear counterstaining was performed with dapi. Whole-slide digital images of the stained sections were generated in a VS200 slidescanner (Evident).

Sections of 3 µm thickness were cut and stained with Periodic Acid-Schiff (PAS, Sigma-Aldrich) and used to semi-quantitatively score mesangial expansion in 20 randomly selected glomeruli as described previously[14]. The observer scoring the mesangial expansion was unaware of the treatment type.

For immunohistochemical staining for CD45, kidney sections were deparaffinised, rehydrated and underwent heat induced epitope retrieval in CC1 buffer (Roche) at 95°C for 15 min. Endogenous peroxidase was blocked with 0.5% H_2O_2 in TBS. Unspecific binding of the primary antibody was blocked with TNB. Subsequently, sections were incubated for 1 hours at 37C with rabbit anti-CD45 antibody (cat. no. ab10558, Abcam), diluted in antibody diluent (Roche). The primary antibody was detected with HRP-labelled OmniMap anti-rabbit polymer (Roche).

Immunohistochemical staining of aSMA was done in a comparable way, with a rabbit anti-aSMA antibody (cat. no. ab124964, Abcam), except that anti-rabbit HQ(Roche) followed by HRP-labelled anti-HQ (Roche) was used for detection of the primary antibody.

Immunohistochemical staining of KIM-1 was done with a goat-anti-mouse KIM-1 antibody was used (cat. no. AF1817, R&D Systems). Staining was done in a comparable way, except that heat-induced epitope retrieval was performed in Tris-EGTA buffer, pH 9, at 95C for 15 min, and the primary antibody was detected with a biotinylated donkey-anti-goat antibody (Jackson Immunoresearch), followed by the Vectastain ABC complex (Vector), diluted in TNB (Roche).

Immunohistochemical staining of collagen 3 was performed with a goat anti-type III collagen antibody (cat. no. 1330-01, Southern Biotech) essentially as described above for KIM-1.

In all stainings purple chromogen was used for visualization and all slides were finally counterstained with haematoxylin and cover glasses mounted with Pertex. Digital whole slide images were generated on a Nanozoomer 2.0 HT slide scanner (Hamamatsu).

Image analysis of in situ hybridization assays (all based on flourescent detection of signals) was performed with the softwares HALO (Indica) and VIS (Visiopharm). In sections stained for Agtr1a and Spp1, 25-30 glomeruli were randomly selected in each kidney section and outlined as region of interest in the software HALO. The fractional area with positive staining for Agtr1a or Spp1 and the mean intensity of the positively stained area was then quantified, and by multiplying the fractional area with the mean intensity of the stained area, the standardized signal

for Agtr1a or Spp1 expression in glomeruli was calculated for each animal. A similar approach was used for quantification of Ren1, Agtr1a and Spp1 in the manually outlined kidney cortex for each animal, except that this was done in the software VIS. Quantification of CD45, KIM-1, aSMA and collagen 3, which were all detected with chromogenic IHC assays, was done in VIS, by quantifying the fractional area with positive staining for each animal (the so-called area fraction).

Statistical analysis of histology data was performed as a Brown-Forsythe one-side analysis of variance, followed by pairwise comparison of each group to the vehicle-treated group of NTN mice in parallel t-tests, with Dunnetts correction for multiple parallel tests. Data for Ren1 expression and Spp1 expression in glomeruli were log-transformed with the natural logarithm prior to analysis. The number of animals per group included in the ISH and IHC assays are listed in Supplemental Table 4 below. When ISH and IHC data were compared to Visium ST data for validation (Supplemental Table 3 below), the comparisons were based on the 5 animals per group where Visium ST data, ISH data and IHC data were available.

Filtration slit density

The filtration slit density (FSD) describes the ration between the length of the filtration slit and the surface area. A high value describes an intact morphology whereas a low value shows an effacement. The analysis was performed by the CRO; NIPOKA (Germany) according to the method described in detail previously[12], using a rabbit anti-podocin antibody (IBL, catalog number JP29040) and a mouse anti-Synaptopodin antibody (Progen, catalog number 61094). Kidney slices were obtained from formalin-fixed and paraffin-embedded tissue that were stained for podocin that makes the filtration slit visible. Image analysis was performed to detect and measure the filtration slit and the FSD is calculated. A total of 20 randomly selected glomeruli were analysed per sample.

Supplemental Results

Gene set enrichment in proximal tubules

GSEA in PT appeared fully comparable to the pattern in glomeruli and areas with VSMC: Multiple immune functions and some fibrosis-related functions were positively enriched in vehicle-treated NTN animals compared to healthy, and this was partially reversed in animals treated with semaglutide or enalapril (Supplemental Table 2). Of single genes, we were particularly interested in

Havcr1 (alias: KIM-1), which is a marker of tubular damage. Expression of this gene increased ≈11-fold in vehicle-treated NTN mice relative to healthy (where expression is minimal), and was significantly decreased after treatment with enalapril, but displayed <1.2-fold change in expression in semaglutide-treated animals. This was in good agreement with the KIM-1 IHC results described in the main text. Furthermore, previous studies have reported a natriuretic effect of GLP-1RA agonist treated via decreased activity of Na^+/H^+ exchanger 3 (gene name: Slc9a3) in the proximal tubules[15,16]. Interestingly, expression of Slc9a3 was significantly increased with ≈1.8 fold in semaglutide-treated animals, and only ≈1.5 fold in enalapril-treated animals.

Semaglutide and enalapril had moderate effects on expression of genes in the renin-angiotensin-aldosterone system

In previous studies treatment with GLP-1RA influenced expression of key genes in RAAS, and we therefore explored their expression in all kidney compartments (Supplemental Figure 8). The clearest effect was significantly increased expression of Ren1 in the glomerular compartment after treatment with semaglutide as well as enalapril. The other genes were expressed at low levels in the kidney, except for Ace, which was expressed at somewhat higher levels in the OSOM. However, expression levels of any of these genes were not influenced significantly by treatment with semaglutide or enalapril. We confirmed this pattern with ISH for Ren1 and Agtr1a; expression of Ren1 was significantly increased in animals treated with semaglutide and animals treated with enalapril (most pronounced in enalapril-treated animals) (Figure 5A), and we observed no significant effects of treatment on expression levels of Agtr1a (Figure 5B).

Supplemental Tables

Supplemental Table 1. DEA genes identified in the ST data.

Supplemental Table 2. GSEA of the ST data.

Supplemental Table 3. Comparison of relative differences between groups (fold changes) observed in Visium spatial transcriptomics data to relative differences observed with ISH or IHC

	Vehicle vs. Healthy		Sema vs. Vehicle		Enalapril vs. Vehicle	
Kidney cortex:	FC by Visium	FC by ISH or IHC	FC by Visium	FC by ISH or IHC	FC by Visium	FC by ISH or IHC
Ren1 (ISH)	0.20	0.99	10.1	1.07	106.2	4.26
Agtr1a (ISH)	1.13	1.44	1.66	0.83	1.24	1.24
Spp1 (ISH)	2.50	2.33	0.43	0.61	0.43	0.71
Havcr1/KIM-1 (IHC)	29.0	412.2	0.72	0.26	0.12	0.02
Col3a1 (IHC)	3.51	3.76	0.51	0.77	0.31	0.38
Acta2/aSMA (IHC)	1.47	2.62	1.36	0.93	0.90	0.60
Ptprc/CD45 (IHC)	2.46	6.10	0.45	0.39	0.63	0.19
Glomeruli:						
Agtr1a (ISH)	1.33	1.53	1.59	0.95	1.20	1.22
Spp1 (ISH)	2.17	4.15	0.42	0.52	0.49	0.52
Mesangial matrix proteins†	2.38	mean MES=1.04 vs. mean MES=0	0.68	mean MES=0.15 vs. mean MES=1.04	0.54	mean MES=0.04 vs. mean MES=1.04

FC=Mean fold change

†Geometric mean of fold changes in Visium assay for the following genes encoding mesangial matrix proteins and glycoproteins: Fn1, Col4a1, Lama2, Nid1, Bgn, Dcn.

Supplemental Table 4. Number of animals per group included in ISH and IHC assays.

Assay	Healthy controls	NTN + Vehicle	NTN + Sema	NTN + Enalapril
All ISH assays	7	9	9	7
CD45 IHC	10	10	11	9
Filtration slit density	6	10	11	9
PAS stain (for MES)	10	10	11	9
KIM-1 IHC	11	10	11	9
aSMA IHC	11	10	11	9
Collagen 3 IHC	11	10	11	9

Supplemental Figures

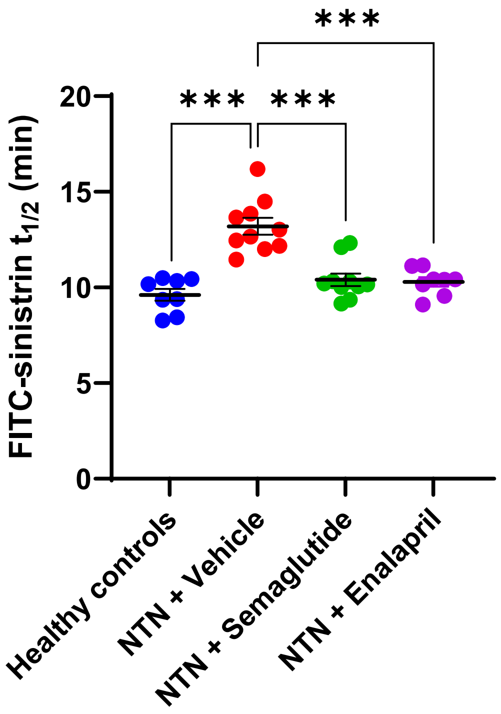

Supplemental Figure 1. Half-life of FITC-sinistrin. Symbols are observations from individual animals, horizontal lines and error bars indicate mean±SEM. *** indicate P<0.001 in the comparisons indicated on the figure.

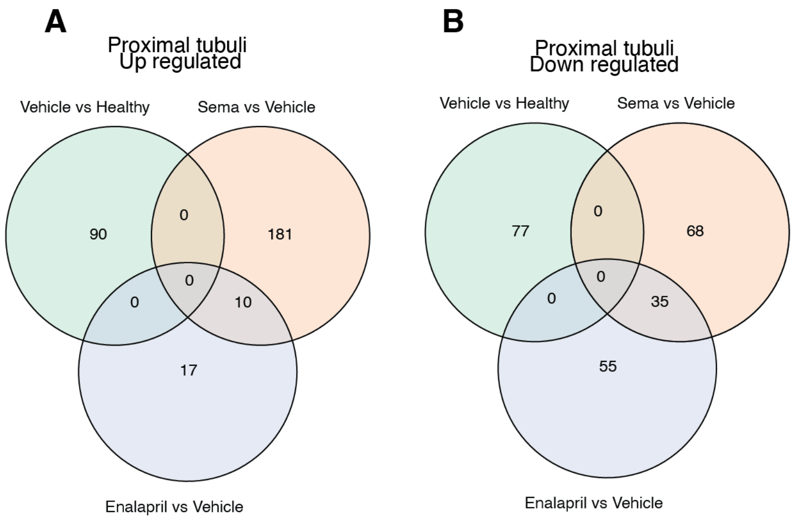

Supplemental Figure 2. Venn diagram of genes regulated in PT, when vehicle-treated NTN mice, semaglutide-treated NTN mice and enalapril-treated NTN mice were compared to their corresponding control groups. A: Up regulated genes. B: Down regulated genes.

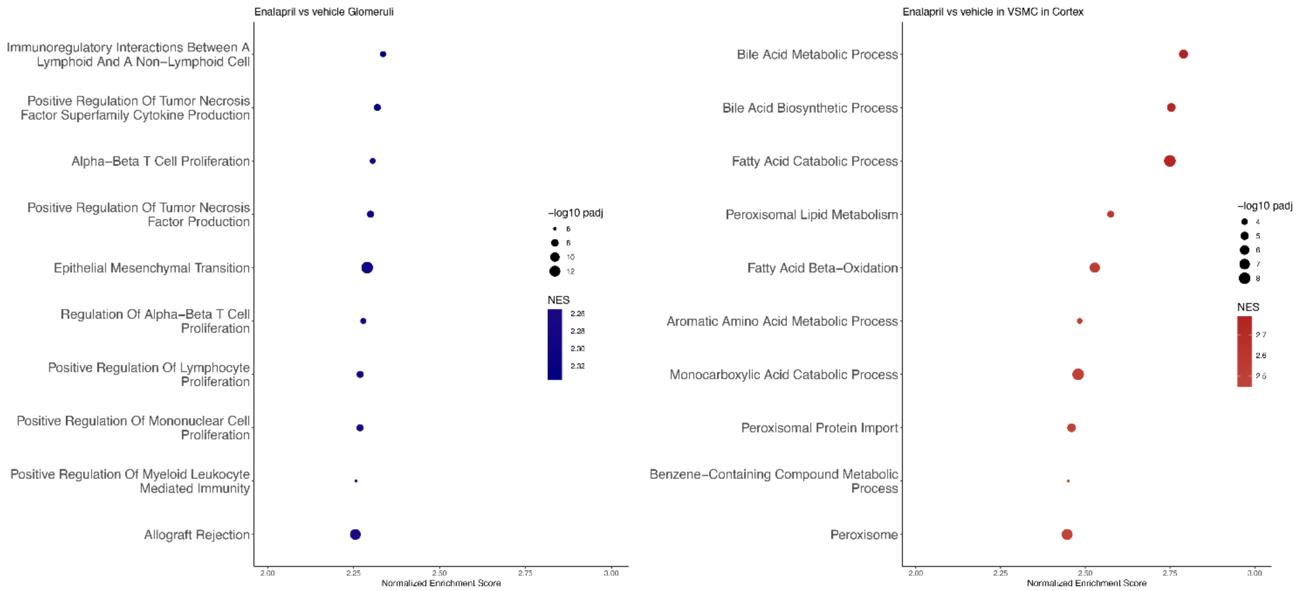

Supplemental Figure 3. Top 10 significant enriched gene sets in Enalapril vs vehicle. Compartments are described on the figure.

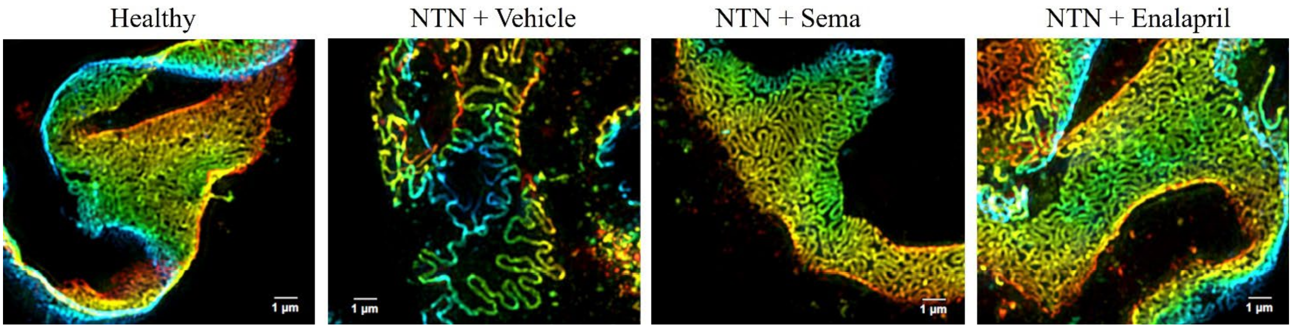

Supplemental Figure 4: Representative images of podocin staining.
Immunohistochemical staining of podocin was used to quantify filtration slit density. The images of the podocin staining has been color-coded according to the position of the staining in the z-axis. The images represent the four groups in the experiment, as indicated in the legend above each image.

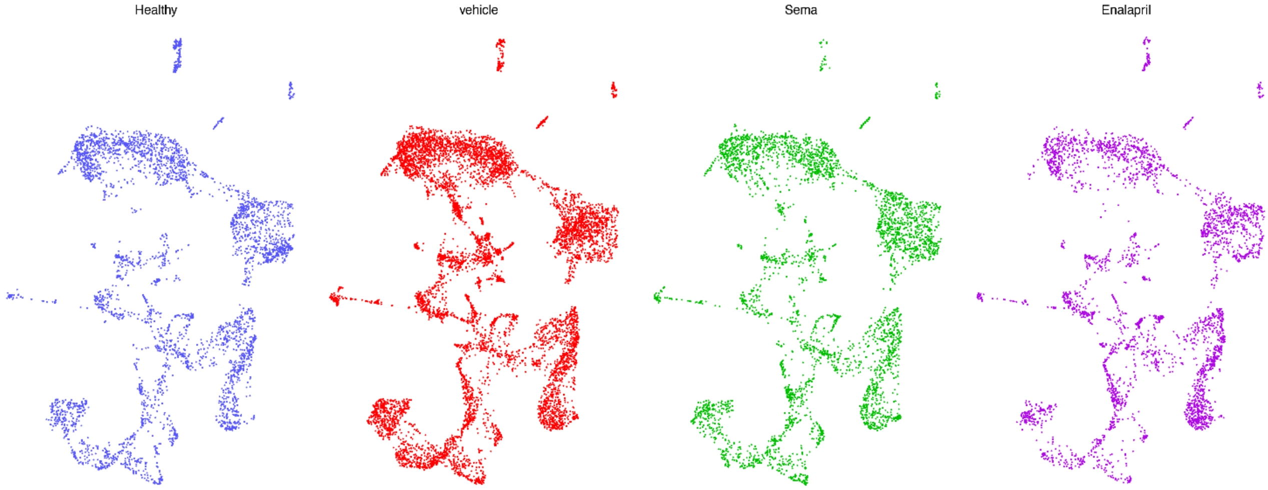

Supplemental Figure 5. UMAP of the snRNAseq data colored by treatment.

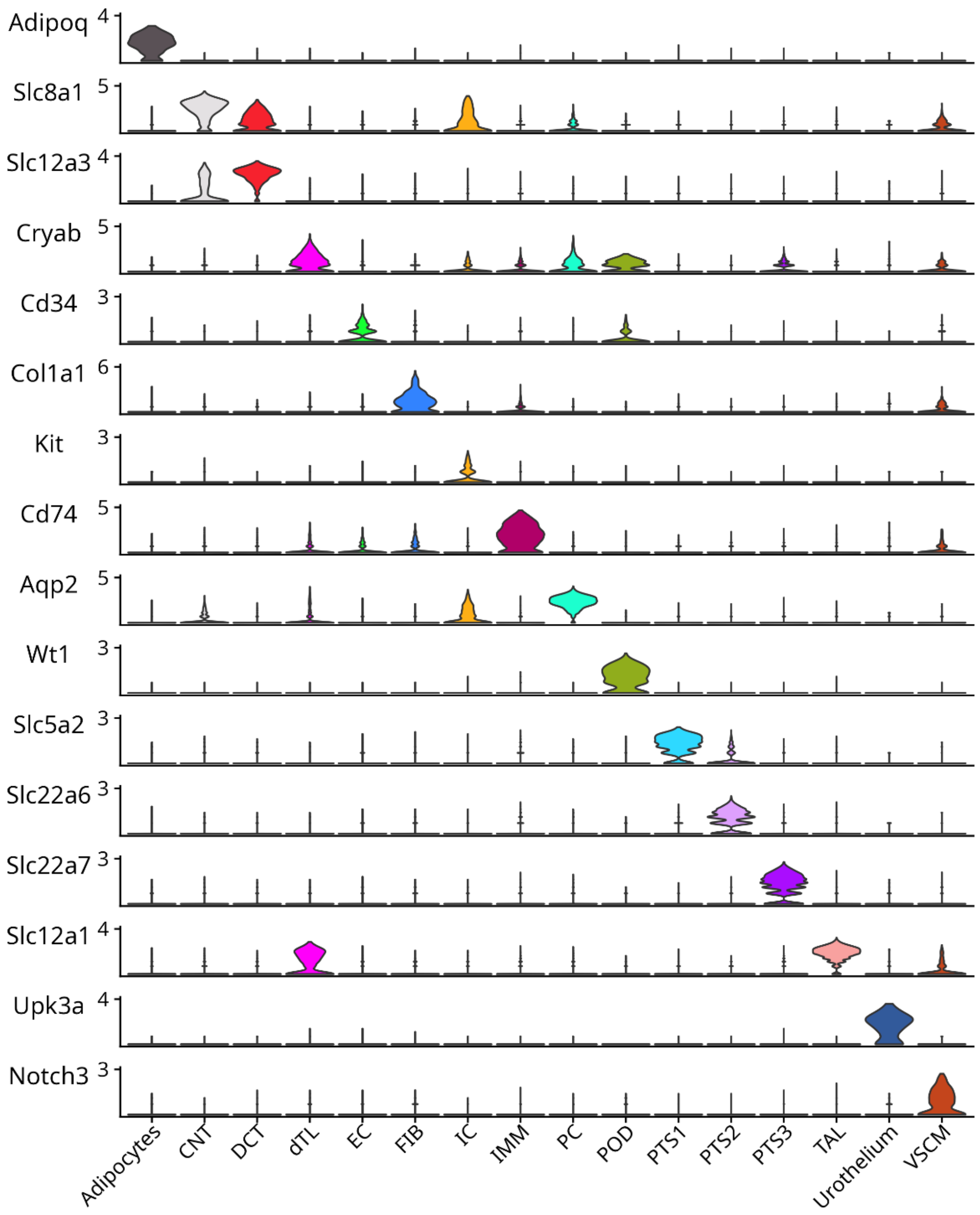

Supplemental Figure 6. Key markers showing cell type annotation in the snRNAseq dataset.

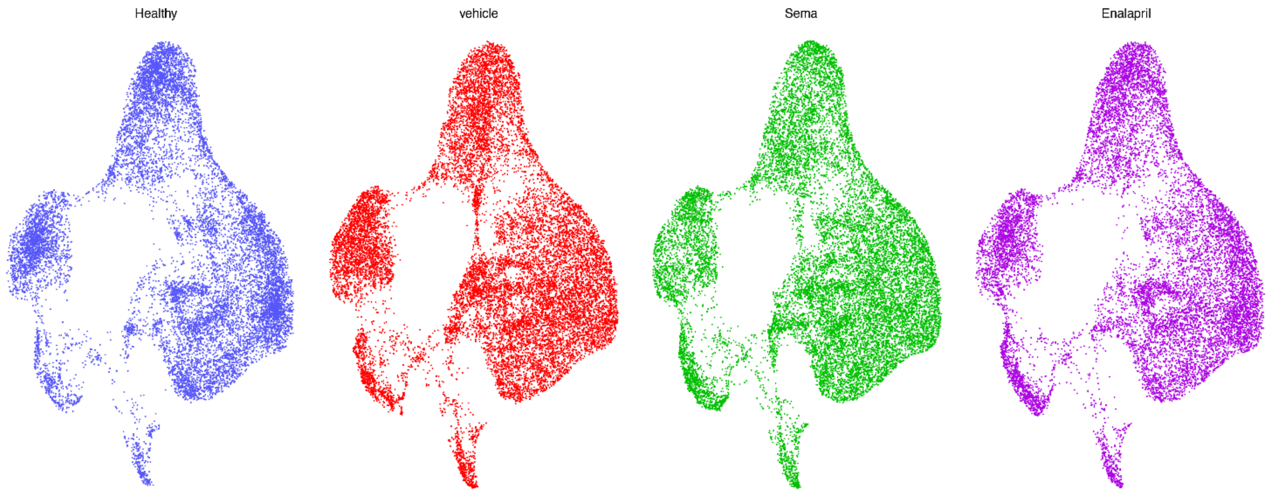

Supplemental Figure 7. UMAP of the ST dataset colored by treatment.

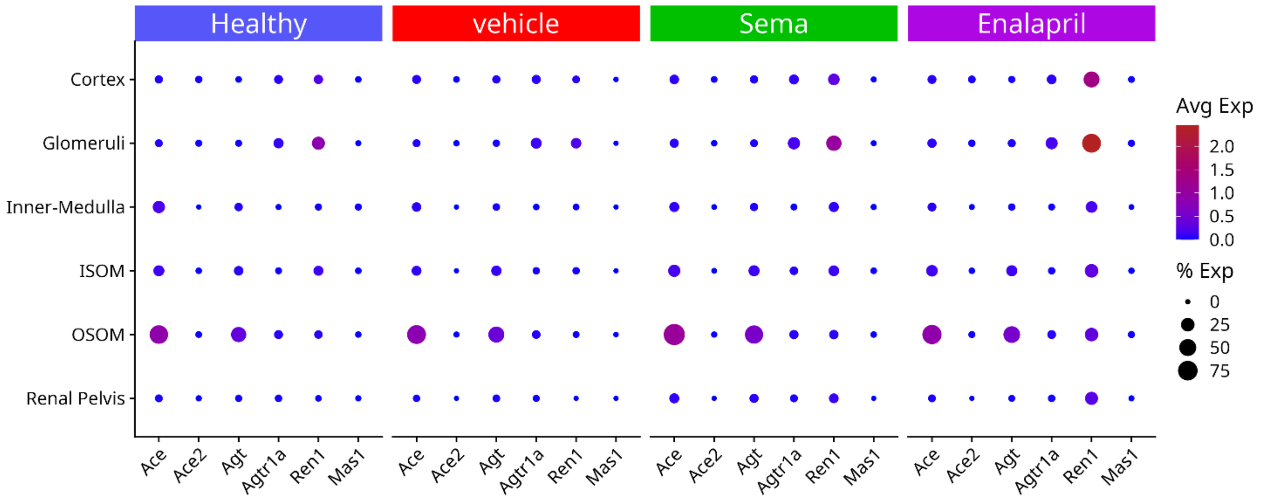

Supplemental Figure 8. Expression levels of key genes belonging to the RAAS in the different kidney compartments for each experimental group.

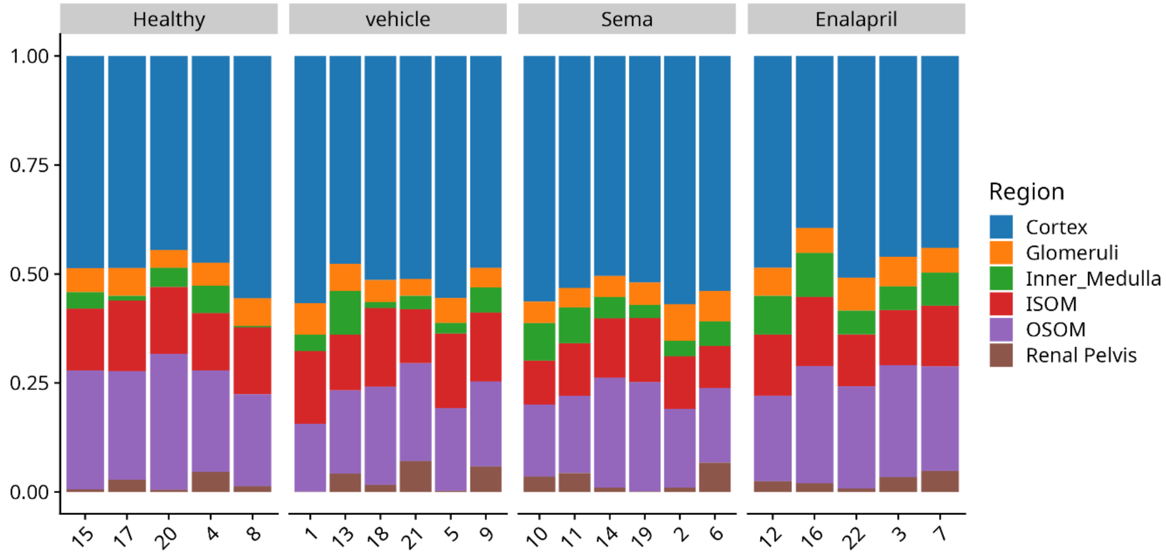

Supplemental Figure 9. Relative abundance of kidney compartments in each individual animal.

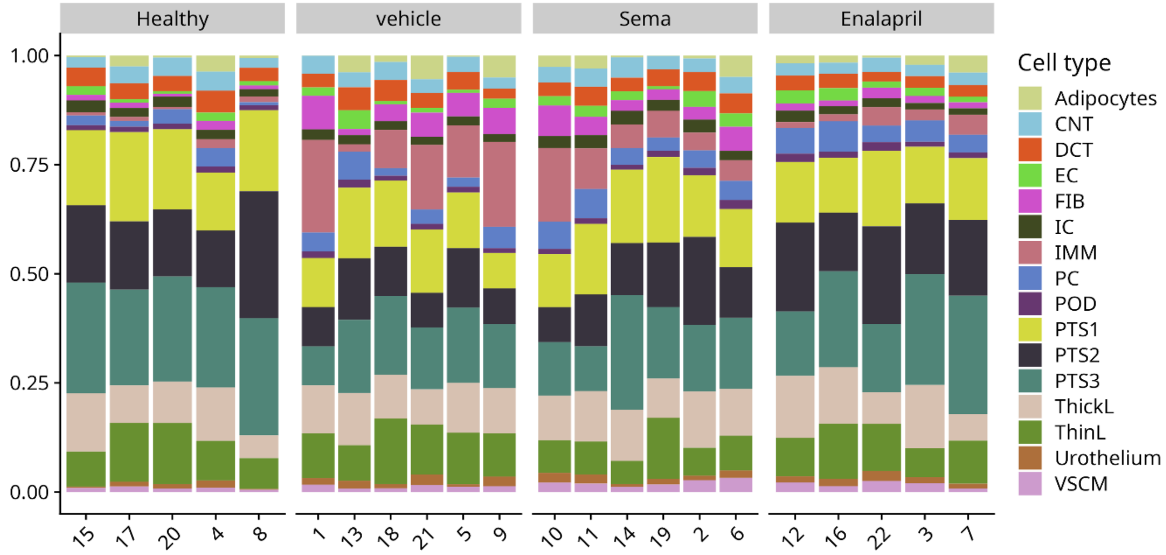

Supplemental Figure 10. Relative abundance of cell types in each individual animal.

Supplemental references

1. Wolock SL, Lopez R, Klein AM: Scrublet: Computational Identification of Cell Doublets in Single-Cell Transcriptomic Data. *Cell Syst* Apr 24 2019;8(4):281-291.e289. doi:10.1016/j.cels.2018.11.005
2. Hafemeister C, Satija R: Normalization and variance stabilization of single-cell RNA-seq data using regularized negative binomial regression. *Genome Biol* Dec 23 2019;20(1):296. doi:10.1186/s13059-019-1874-1
3. Korsunsky I, Millard N, Fan J, Slowikowski K, Zhang F, Wei K, Baglaenko Y, Brenner M, Loh PR, Raychaudhuri S: Fast, sensitive and accurate integration of single-cell data with Harmony. *Nat Methods* Dec 2019;16(12):1289-1296. doi:10.1038/s41592-019-0619-0
4. Lake BB, Menon R, Winfree S, Hu Q, Melo Ferreira R, Kalhor K, Barwinska D, Otto EA, Ferkowicz M, Diep D, Plongthongkum N, Knoten A, Urata S, Mariani LH, Naik AS, Eddy S, Zhang B, Wu Y, Salamon D, Williams JC, Wang X, Balderrama KS, Hoover PJ, Murray E, Marshall JL, Noel T, Vijayan A, Hartman A, Chen F, Waikar SS, Rosas SE, Wilson FP, Palevsky PM, Kiryluk K, Sedor JR, Toto RD, Parikh CR, Kim EH, Satija R, Greka A, Macosko EZ, Kharchenko PV, Gaut JP, Hodgin JB, Consortium K, Eadon MT, Dagher PC, El-Achkar TM, Zhang K, Kretzler M, Jain S: An atlas of healthy and injured cell states and niches in the human kidney. *Nature* Jul 2023;619(7970):585-594. doi:10.1038/s41586-023-05769-3
5. Miao Z, Balzer MS, Ma Z, Liu H, Wu J, Shrestha R, Aranyi T, Kwan A, Kondo A, Pontoglio M, Kim J, Li M, Kaestner KH, Susztak K: Single cell regulatory landscape of the mouse kidney highlights cellular differentiation programs and disease targets. *Nat Commun* Apr 15 2021;12(1):2277. doi:10.1038/s41467-021-22266-1
6. Franzen O, Gan LM, Bjorkegren JLM: PanglaoDB: a web server for exploration of mouse and human single-cell RNA sequencing data. *Database (Oxford)* Jan 1 2019;2019doi:10.1093/database/baz046
7. Xie Z, Bailey A, Kuleshov MV, Clarke DJB, Evangelista JE, Jenkins SL, Lachmann A, Wojciechowicz ML, Kropiwnicki E, Jagodnik KM, Jeon M, Ma'ayan A: Gene Set Knowledge Discovery with Enrichr. *Curr Protoc* Mar 2021;1(3):e90. doi:10.1002/cpz1.90
8. Kleshchevnikov V, Shmatko A, Dann E, Aivazidis A, King HW, Li T, Elmentaite R, Lomakin A, Kedlian V, Gayoso A, Jain MS, Park JS, Ramona L, Tuck E, Arutyunyan A, Vento-Tormo R, Gerstung M, James L, Stegle O, Bayraktar OA: Cell2location maps fine-grained cell types in spatial transcriptomics. *Nat Biotechnol* May 2022;40(5):661-671. doi:10.1038/s41587-021-01139-4
9. Moreno J, Gluud LL, Galsgaard ED, Hvid H, Mazzoni G, Das V: Identification of ligand and receptor interactions in CKD and MASH through the integration of single cell and spatial transcriptomics. *PLoS One* 2024;19(5):e0302853. doi:10.1371/journal.pone.0302853
10. Wu T, Hu E, Xu S, Chen M, Guo P, Dai Z, Feng T, Zhou L, Tang W, Zhan L, Fu X, Liu S, Bo X, Yu G: clusterProfiler 4.0: A universal enrichment tool for interpreting omics data. *Innovation (Camb)* Aug 28 2021;2(3):100141. doi:10.1016/j.xinn.2021.100141
11. Jin S, Guerrero-Juarez CF, Zhang L, Chang I, Ramos R, Kuan CH, Myung P, Plikus MV, Nie Q: Inference and analysis of cell-cell communication using

CellChat. *Nat Commun* Feb 17 2021;12(1):1088. doi:10.1038/s41467-021-21246-9

12. Artelt N, Siegerist F, Ritter AM, Grisk O, Schluter R, Endlich K, Endlich N: Comparative Analysis of Podocyte Foot Process Morphology in Three Species by 3D Super-Resolution Microscopy. *Front Med (Lausanne)* 2018;5:292. doi:10.3389/fmed.2018.00292
13. Jensen DM, Skovsted GF, Bonde MFB, Bentzon JF, Rolin B, Franck G, Ougaard MKE, Voetmann LM, Bachmann JC, Uryga A, Pyke C, Kirk RK, Hvid H, Knudsen LB, Lykkesfeldt J, Nyberg M: Semaglutide treatment attenuates vessel remodelling in ApoE-/- mice following vascular injury and blood flow perturbation. *Atheroscler Plus* Aug 2022;49:32-41. doi:10.1016/j.athplu.2022.05.004
14. Ougaard MKE, Kvist PH, Jensen HE, Hess C, Rune I, Sondergaard H: Murine Nephrotoxic Nephritis as a Model of Chronic Kidney Disease. *Int J Nephrol* 2018;2018:8424502. doi:10.1155/2018/8424502
15. Crajoinas RO, Oricchio FT, Pessoa TD, Pacheco BP, Lessa LM, Malnic G, Girardi AC: Mechanisms mediating the diuretic and natriuretic actions of the incretin hormone glucagon-like peptide-1. *Am J Physiol Renal Physiol* Aug 2011;301(2):F355-363. doi:10.1152/ajprenal.00729.2010
16. Thomson SC, Kashkouli A, Singh P: Glucagon-like peptide-1 receptor stimulation increases GFR and suppresses proximal reabsorption in the rat. *Am J Physiol Renal Physiol* Jan 15 2013;304(2):F137-144. doi:10.1152/ajprenal.00064.2012
