# Supplementary material for: Molecular Characterization of the Effect of Glucagon-Like Peptide-1 Receptor Agonist Semaglutide in the Nephrotoxic Serum Nephritis Mouse Model
Source: Kidney360. 2025 Dec 3;7(4):728–40. doi: 10.34067/KID.0000001067 (PMC13134791; doi:10.34067/KID.0000001067)
Supplement: Supplementary file 3 [file kidney360-7-728-s003.pdf]

ASN American Society of Nephrology | JASN Journal of the American Society of Nephrology | KIDNEY360 Accessing Our World From Every Angle | CJASN Clinical Journal of the American Society of Nephrology

ASN Journal Disclosure Form

As per ASN journal policy, I have disclosed any financial relationships or commitments I have held in the past 36 months as included below. I have listed my Current Employer below to indicate there is a relationship requiring disclosure. If no relationship exists, my Current Employer is not listed.

A. Benardeau reports the following:
Employer: Stealth mode biotech startup; Ownership Interest: Bayer AG; and Patents or Royalties: BAYER AG.

I understand that the information above will be published within the journal article, if accepted, and that failure to comply and/or to accurately and completely report the potential financial conflicts of interest could lead to the following: 1) Prior to publication, article rejection, or 2) Post-publication, sanctions ranging from, but not limited to, issuing a correction, reporting the inaccurate information to the authors' institution, banning authors from submitting work to ASN journals for varying lengths of time, and/or retraction of the published work.

Name: Agnes M. Benardeau
Manuscript ID: K360-2025-000468R2
Manuscript Title: "Molecular characterization of the effect of the GLP-1 receptor agonist semaglutide in the nephrotoxic serum nephritis mouse model"
Date of Completion: October 22, 2025
Disclosure Updated Date: October 22, 2025

ASN American Society of Nephrology | JASN Journal of the American Society of Nephrology | KIDNEY360 Accessing Our World From Every Angle | CJASN Clinical Journal of the American Society of Nephrology

ASN Journal Disclosure Form

As per ASN journal policy, I have disclosed any financial relationships or commitments I have held in the past 36 months as included below. I have listed my Current Employer below to indicate there is a relationship requiring disclosure. If no relationship exists, my Current Employer is not listed.

T. Grancharova reports the following:
Employer: Novo Nordisk; and Ownership Interest: Novo Nordisk.

I understand that the information above will be published within the journal article, if accepted, and that failure to comply and/or to accurately and completely report the potential financial conflicts of interest could lead to the following: 1) Prior to publication, article rejection, or 2) Post-publication, sanctions ranging from, but not limited to, issuing a correction, reporting the inaccurate information to the authors' institution, banning authors from submitting work to ASN journals for varying lengths of time, and/or retraction of the published work.

Name: Tanya Grancharova
Manuscript ID: K360-2025-000468R1
Manuscript Title: Molecular characterization of the effect of the GLP-1 receptor agonist semaglutide in the nephrotoxic serum nephritis mouse model
Date of Completion: August 21, 2025
Disclosure Updated Date: August 21, 2025

ASN
American Society of Nephrology
JASN
Journal of the American Society of Nephrology
KIDNEY360
Accessing Our World From Every Angle
CJASN
Clinical Journal of the American Society of Nephrology

ASN Journal Disclosure Form

As per ASN journal policy, I have disclosed any financial relationships or commitments I have held in the past 36 months as included below. I have listed my Current Employer below to indicate there is a relationship requiring disclosure. If no relationship exists, my Current Employer is not listed.

H. Hvid reports the following:
Employer: Novo Nordisk A/S; Ownership Interest: Novo Nordisk A/S; and Research Funding: Novo Nordisk A/S.

I understand that the information above will be published within the journal article, if accepted, and that failure to comply and/or to accurately and completely report the potential financial conflicts of interest could lead to the following: 1) Prior to publication, article rejection, or 2) Post-publication, sanctions ranging from, but not limited to, issuing a correction, reporting the inaccurate information to the authors' institution, banning authors from submitting work to ASN journals for varying lengths of time, and/or retraction of the published work.

Name: Henning Hvid
Manuscript ID: K360-2025-000468R1
Manuscript Title: Molecular characterization of the effect of the GLP-1 receptor agonist semaglutide in the nephrotoxic serum nephritis mouse model
Date of Completion: August 20, 2025
Disclosure Updated Date: August 20, 2025

ASN American Society of Nephrology JASN Journal of the American Society of Nephrology KIDNEY360 Accessing Our World From Every Angle CJASN Clinical Journal of the American Society of Nephrology

ASN Journal Disclosure Form

As per ASN journal policy, I have disclosed any financial relationships or commitments I have held in the past 36 months as included below. I have listed my Current Employer below to indicate there is a relationship requiring disclosure. If no relationship exists, my Current Employer is not listed.

L. Knudsen reports the following:
Employer: Novo Nordisk; Ownership Interest: Novo Nordisk; and Patents or Royalties: Novo Nordisk, named inventor, but full company ownership, no royalities attached.

I understand that the information above will be published within the journal article, if accepted, and that failure to comply and/or to accurately and completely report the potential financial conflicts of interest could lead to the following: 1) Prior to publication, article rejection, or 2) Post-publication, sanctions ranging from, but not limited to, issuing a correction, reporting the inaccurate information to the authors' institution, banning authors from submitting work to ASN journals for varying lengths of time, and/or retraction of the published work.

Name: Lotte Bjerre Knudsen
Manuscript ID: K360-2025-000468R2
Manuscript Title: Molecular characterization of the effect of the GLP-1 receptor agonist semaglutide in the nephrotoxic serum nephritis mouse model
Date of Completion: October 25, 2025
Disclosure Updated Date: October 25, 2025

ASN American Society of Nephrology JASN Journal of the American Society of Nephrology KIDNEY360 Accessing Our World From Every Angle CJASN Clinical Journal of the American Society of Nephrology

ASN Journal Disclosure Form

As per ASN journal policy, I have disclosed any financial relationships or commitments I have held in the past 36 months as included below. I have listed my Current Employer below to indicate there is a relationship requiring disclosure. If no relationship exists, my Current Employer is not listed.

P. Kvist reports the following:
Employer: Novo Nordisk A/S; and Ownership Interest: Novo Nordisk A/S.

I understand that the information above will be published within the journal article, if accepted, and that failure to comply and/or to accurately and completely report the potential financial conflicts of interest could lead to the following: 1) Prior to publication, article rejection, or 2) Post-publication, sanctions ranging from, but not limited to, issuing a correction, reporting the inaccurate information to the authors' institution, banning authors from submitting work to ASN journals for varying lengths of time, and/or retraction of the published work.

Name: Peter Helding Kvist
Manuscript ID: K360-2025-000468R2
Manuscript Title: Molecular characterization of the effect of the GLP-1 receptor agonist semaglutide in the nephrotoxic serum nephritis mouse model
Date of Completion: October 9, 2025
Disclosure Updated Date: October 9, 2025

ASN
American Society of Nephrology
JASN Journal of the American Society of Nephrology
KIDNEY360 Accessing Our World From Every Angle
CJASN Clinical Journal of the American Society of Nephrology

ASN Journal Disclosure Form

As per ASN journal policy, I have disclosed any financial relationships or commitments I have held in the past 36 months as included below. I have listed my Current Employer below to indicate there is a relationship requiring disclosure. If no relationship exists, my Current Employer is not listed.

J. Moreno Martinez reports the following:
Employer: Novo Nordisk A/S; and Ownership Interest: Novo Nordisk A/S.

I understand that the information above will be published within the journal article, if accepted, and that failure to comply and/or to accurately and completely report the potential financial conflicts of interest could lead to the following: 1) Prior to publication, article rejection, or 2) Post-publication, sanctions ranging from, but not limited to, issuing a correction, reporting the inaccurate information to the authors' institution, banning authors from submitting work to ASN journals for varying lengths of time, and/or retraction of the published work.

Name: Jaime T Moreno Martinez
Manuscript ID: K360-2025-000468R1
Manuscript Title: Molecular characterization of the effect of the GLP-1 receptor agonist semaglutide in the nephrotoxic serum nephritis mouse model
Date of Completion: August 20, 2025
Disclosure Updated Date: August 20, 2025

ASN
American Society of Nephrology
JASN
Journal of the American Society of Nephrology
KIDNEY360
Accessing Our World From Every Angle
CJASN
Clinical Journal of the American Society of Nephrology

ASN Journal Disclosure Form

As per ASN journal policy, I have disclosed any financial relationships or commitments I have held in the past 36 months as included below. I have listed my Current Employer below to indicate there is a relationship requiring disclosure. If no relationship exists, my Current Employer is not listed.

M. Østergaard reports the following:
Employer: Novo Nordisk (DK): current employer, from Sept 2023. Past employers: Tribune Therapeutics (NO), Nov 2022-Aug 2023; CSL Vifor (CH), Apr-Aug 2022; Gubra (DK), Oct 2017-Mar 2022.

I understand that the information above will be published within the journal article, if accepted, and that failure to comply and/or to accurately and completely report the potential financial conflicts of interest could lead to the following: 1) Prior to publication, article rejection, or 2) Post-publication, sanctions ranging from, but not limited to, issuing a correction, reporting the inaccurate information to the authors' institution, banning authors from submitting work to ASN journals for varying lengths of time, and/or retraction of the published work.

Name: Mette Viberg Østergaard
Manuscript ID: K360-2025-000468R2
Manuscript Title: Molecular characterization of the effect of the GLP-1 receptor agonist semaglutide in the nephrotoxic serum nephritis mouse model
Date of Completion: October 23, 2025
Disclosure Updated Date: October 23, 2025

ASN American Society of Nephrology | JASN Journal of the American Society of Nephrology | KIDNEY360 Accessing Our World From Every Angle | CJASN Clinical Journal of the American Society of Nephrology

ASN Journal Disclosure Form

As per ASN journal policy, I have disclosed any financial relationships or commitments I have held in the past 36 months as included below. I have listed my Current Employer below to indicate there is a relationship requiring disclosure. If no relationship exists, my Current Employer is not listed.

M. Ougaard reports the following:
Employer: Gubra

I understand that the information above will be published within the journal article, if accepted, and that failure to comply and/or to accurately and completely report the potential financial conflicts of interest could lead to the following: 1) Prior to publication, article rejection, or 2) Post-publication, sanctions ranging from, but not limited to, issuing a correction, reporting the inaccurate information to the authors' institution, banning authors from submitting work to ASN journals for varying lengths of time, and/or retraction of the published work.

Name: Maria Katarina Ougaard
Manuscript ID: K360-2025-000468R1
Manuscript Title: Molecular characterization of the effect of the GLP-1 receptor agonist semaglutide in the nephrotoxic serum nephritis mouse model
Date of Completion: August 20, 2025
Disclosure Updated Date: August 20, 2025

ASN
American Society of Nephrology
JASN
Journal of the American Society of Nephrology
KIDNEY360
Accessing Our World From Every Angle
CJASN
Clinical Journal of the American Society of Nephrology

ASN Journal Disclosure Form

As per ASN journal policy, I have disclosed any financial relationships or commitments I have held in the past 36 months as included below. I have listed my Current Employer below to indicate there is a relationship requiring disclosure. If no relationship exists, my Current Employer is not listed.

C. Pyke reports the following:
Employer: Novo Nordisk A/S; and Ownership Interest: Novo Nordisk A/S.

I understand that the information above will be published within the journal article, if accepted, and that failure to comply and/or to accurately and completely report the potential financial conflicts of interest could lead to the following: 1) Prior to publication, article rejection, or 2) Post-publication, sanctions ranging from, but not limited to, issuing a correction, reporting the inaccurate information to the authors' institution, banning authors from submitting work to ASN journals for varying lengths of time, and/or retraction of the published work.

Name: Charles C Pyke
Manuscript ID: K360-2025-000468R2
Manuscript Title: Molecular characterization of the effect of the GLP-1 receptor agonist semaglutide in the nephrotoxic serum nephritis mouse model
Date of Completion: October 23, 2025
Disclosure Updated Date: October 23, 2025

ASN American Society of Nephrology | JASN Journal of the American Society of Nephrology | KIDNEY360 Accessing Our World From Every Angle | CJASN Clinical Journal of the American Society of Nephrology

ASN Journal Disclosure Form

As per ASN journal policy, I have disclosed any financial relationships or commitments I have held in the past 36 months as included below. I have listed my Current Employer below to indicate there is a relationship requiring disclosure. If no relationship exists, my Current Employer is not listed.

R. Zdravkovic reports the following:
Employer: Novo Nordisk A/S; and Ownership Interest: Novo Nordisk A/S;.

I understand that the information above will be published within the journal article, if accepted, and that failure to comply and/or to accurately and completely report the potential financial conflicts of interest could lead to the following: 1) Prior to publication, article rejection, or 2) Post-publication, sanctions ranging from, but not limited to, issuing a correction, reporting the inaccurate information to the authors' institution, banning authors from submitting work to ASN journals for varying lengths of time, and/or retraction of the published work.

Name: Regitze Dalsgaard D Zdravkovic
Manuscript ID: K360-2025-000468R2
Manuscript Title: Molecular characterization of the effect of the GLP-1 receptor agonist semaglutide in the nephrotoxic serum nephritis mouse model
Date of Completion: October 24, 2025
Disclosure Updated Date: October 24, 2025
